# Deep-learning-based survival prediction of patients with cutaneous malignant melanoma

**DOI:** 10.3389/fmed.2023.1165865

**Published:** 2023-03-27

**Authors:** Hai Yu, Wei Yang, Shi Wu, Shaohui Xi, Xichun Xia, Qi Zhao, Wai-kit Ming, Lifang Wu, Yunfeng Hu, Liehua Deng, Jun Lyu

**Affiliations:** ^1^Department of Dermatology, The First Affiliated Hospital of Jinan University and Jinan University Institute of Dermatology, Guangzhou, China; ^2^Office of Drug Clinical Trial Institution, The First Affiliated Hospital of Jinan University, Guangzhou, China; ^3^School of Mechatronical Engineering, Guangdong Polytechnic Normal University, Guangzhou, China; ^4^Institute of Biomedical Transformation, Jinan University, Guangzhou, China; ^5^Cancer Centre, Faculty of Health Sciences, University of Macau, Macau, China; ^6^Department of Infectious Diseases and Public Health, Jockey Club College of Veterinary Medicine and Life Sciences, City University of Hong Kong, Hong Kong, China; ^7^Department of Dermatology, The Fifth Affiliated Hospital of Jinan University, Heyuan, China; ^8^Department of Clinical Research, The First Affiliated Hospital of Jinan University, Guangzhou, China; ^9^Guangdong Provincial Key Laboratory of Traditional Chinese Medicine Informatization, Guangzhou, China

**Keywords:** DeepSurv, cutaneous malignant melanoma, neural network, survival prediction, SEER

## Abstract

**Background:**

This study obtained data on patients with cutaneous malignant melanoma (CMM) from the Surveillance, Epidemiology, and End Results (SEER) database, and used a deep learning and neural network (DeepSurv) model to predict the survival rate of patients with CMM and evaluate its effectiveness.

**Methods:**

We collected information on patients with CMM between 2004 and 2015 from the SEER database. We then randomly divided the patients into training and testing cohorts at a 7:3 ratio. The likelihood that patients with CMM will survive was forecasted using the DeepSurv model, and its results were compared with those of the Cox proportional-hazards (CoxPH) model. The calibration curves, time-dependent area under the receiver operating characteristic curve (AUC), and concordance index (C-index) were used to assess the prediction abilities of the model.

**Results:**

This study comprised 37,758 patients with CMM: 26,430 in the training cohort and 11,329 in the testing cohort. The CoxPH model demonstrated that the survival of patients with CMM was significantly influenced by age, sex, marital status, summary stage, surgery, radiotherapy, chemotherapy, postoperative lymph node dissection, tumor size, and tumor extension. The C-index of the CoxPH model was 0.875. We also constructed the DeepSurv model using the data from the training cohort, and its C-index was 0.910. We examined how well the aforementioned two models predicted outcomes. The 1-, 3-, and 5-year AUCs were 0.928, 0.837, and 0.855, respectively, for the CoxPH model, and 0.971, 0.947, and 0.942 for the DeepSurv model. The DeepSurv model presented a greater predictive effect on patients with CMM, and its reliability was better than that of the CoxPH model according to both the AUC value and the calibration curve.

**Conclusion:**

The DeepSurv model, which we developed based on the data of patients with CMM in the SEER database, was found to be more effective than the CoxPH model in predicting the survival time of patients with CMM.

## Background

Cutaneous malignant melanoma (CMM) is a cancerous tumor that affects the skin and mucous membranes. It is currently the most prevalent tumor in the US. Melanoma has been estimated to develop in 1 of 63 Americans during their lifetimes (1, 2). Among those aged 40–75 years, it affects males approximately three times more frequently than it does females, while males of all ages are around 1.5 times more likely than females to develop it. Young and middle-aged persons can develop CMM, and the median age at diagnosis is 57 years (2–4). CMM incidence has been seen to increase linearly among those aged 25–50 years, and accounts for 65% of all skin cancer deaths (5, 6). Early detection and treatment of CMM can increase the 5-year overall survival rate to 95%. However, after metastasis, only 5% of patients with CMM survive over the long term (7, 8). Of course, the prognosis of different patients is affected by many factors, and the survival rate is also different, even very different. The TNM staging system of the American Joint Committee on Cancer is currently the primary basis for therapy decision-making in CMM (9). Wide local excision within 30 days of the first biopsy was found to decrease mortality in patients at stage I, but not at stage II or III (10). Clinical research has revealed that even if they receive the same treatment, different patients with CMM at the same stage have been found to have varying therapeutic results and survival rates. Death rate from metastatic melanoom is still high in spite of immunecheckpoint therapy (11). Age, sex, race, tumor site, and the therapy method are all risk factors that have been linked to the prognosis of patients with CMM (12–15). The prognosis of patients with CMM in the US is influenced by numerous variables, the most important of which is tumor stage. In order to estimate the risk factors for and survival rate of patients with CMM, new assessment techniques are required that can concurrently consider various influencing factors.

Cox proportional-hazards (CoxPH) models and nomograms are some of the survival analysis models that are currently often employed in clinical practice (16). However, these model are semiparametric linear proportional-hazard models, which may ignore the variations in the impact of factors that affect patients at different times when making personalized treatment recommendations, which oversimplifies the relationship between predictors and patient prognosis (17–19). Reliable survival predictions that take into account the non-linear relationship between predictions and predictors require more complex and precise algorithms in survival models. Deep learning and neural network models have recently been applied to clinical medicine. These models have successfully tackled multifactor and nonlinear mechanisms. The CoxPH deep neural network (DeepSurv) model created by Katzman et al. using deep neural networks and CoxPH produced positive outcomes when utilized as a tool for clinical personalized treatment (20).

The goal of this study was to construct a DeepSurv model using the data of patients with CMM in the Surveillance, Epidemiology, and End Results (SEER) database to predict the mortality of CMM and provide a theoretical foundation for its treatment.

## Materials and methods

### Data filtering criteria

**Figure 1:**
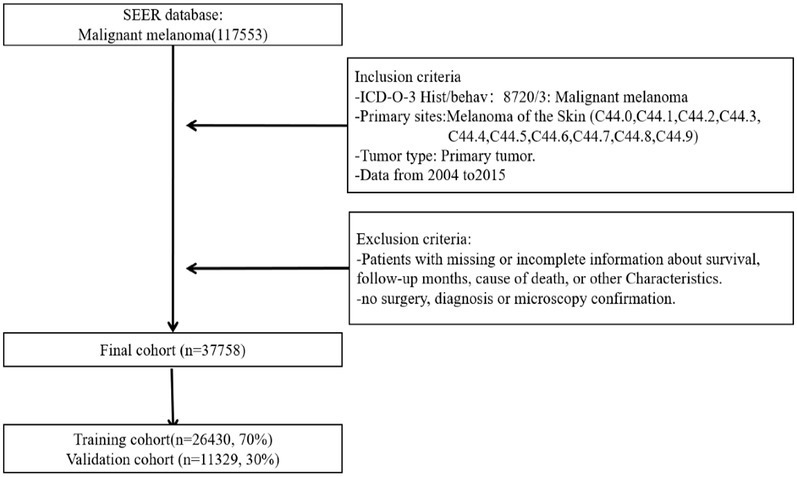
Flow chart of patient selection.

SEER*Stat software (version 8.4.0.1) was used to screen patients with CMM (21, 22). We selected patients with primary tumors diagnosed as CMM according to the third edition of the International Classification of Diseases for Oncology during 2004–2015 (primary site codes C44.0–C44.9). Patients with incomplete basic information and nonprimary tumors were excluded. Finally, 37,758 patients with CMM were enrolled. There was no need to obtained signed informed consents or ethics committee approval because all data in the SEER database other than patient identity information is accessible to the general public. The flow chart of patient selection is depicted in Figure 1.

### Patient information classification criteria

The examined variables included age, sex, race, marital status, tumor size, tumor extension, TNM stage, summary stage, surgery status, radiotherapy status, chemotherapy status, lymph node dissection after surgery (Reg LN Sur), surgery of primary site, sequence of radiotherapy (Rad Seq), and income. The median follow-up period was 85.03 months (range 1–191 months). White, black, and other were the three categories for race, and married, single, and other were those for marital status. The three main tumor locations were the head, face, and neck, the trunk, and the limbs. Reg LN Sur was divided into postoperative lymph node dissection and no lymph node examination or removal. Rad Seq was divided into no radiotherapy, radiotherapy before surgery, radiotherapy during surgery, radiotherapy after surgery, and radiotherapy both before and after surgery.

### DeepSurv model design

**Figure 2:**
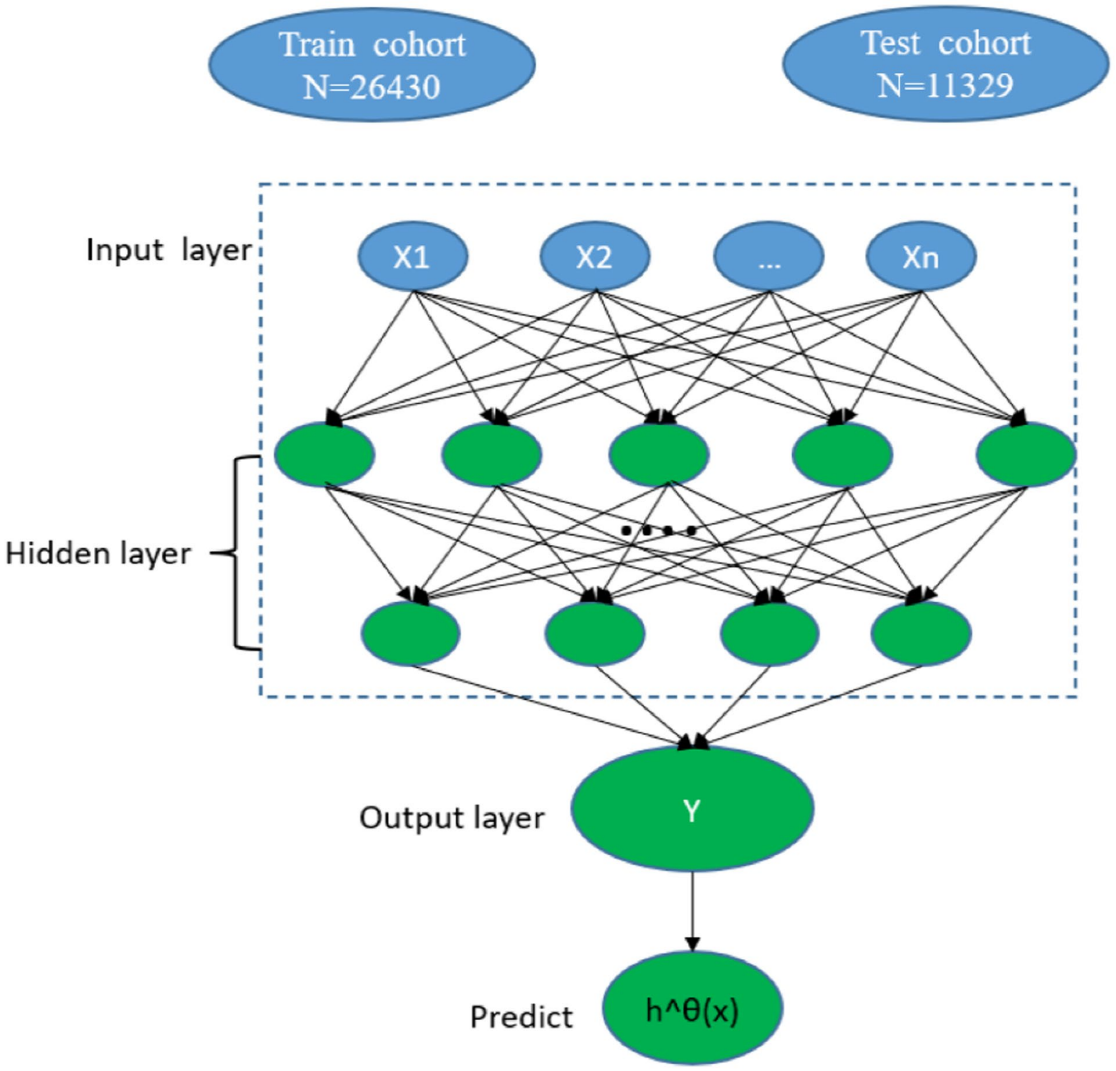
Diagram of the deep learning procedure.

The DeepSurv model is a feedforward neural network with three layers (input, hidden, and output) and comprises many simulated neurons. The baseline data of the patients (*x*) serves as the input layer, a fully linked nonlinear activation function and dropout serve as the hidden layer, and the estimated risk value serves as the output layer *h*^θ(*x*) (20) (Figure 2). Based on the PyTorch deep learning framework, our model primarily used pycox to execute neural network calculations. The model forecasts the effects of different clinical characteristics on patient survival to produce a risk value. We divided the patients with CMM into training and testing cohorts. A neural network with seven layers was first constructed for the DeepSurv model using the training cohort data. The model was subsequently used to perform a survival analysis on the patients with CMM testing cohort. Finally, the concordance index (C-index), calibration curve, and receiver operating characteristic (ROC) curve were used to compare the discrimination, calibration, and effectiveness of the DeepSurv models.

### Statistical analysis

Categorical variables are expressed as percentages, and continuous variables as median and interquartile values. R software (version 4.0.1) and Python software (version 3.7.6) were used for the CoxPH and DeepSurv models, respectively. The pandas, matplotlib.pyplot, and NumPy libraries were used in Python for data processing, model training, and other procedures (23). We used the PyTorch deep learning framework and the pycox module to construct a deep learning neural network. Statistical results were considered significant at *p* < 0.05.

## Results

### Basic information of patients

**Figure 3:**
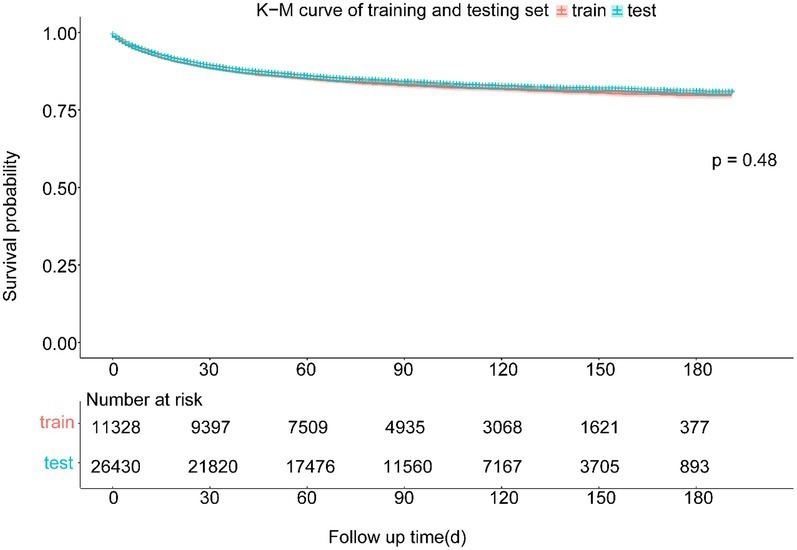
Kaplan–Meier curve of training and testing cohort. There was no statistically significant difference between the survival of training and testing cohort in the log-rank test (*p* = 0.48).

The study included 37,758 patients with CMM, who were divided into training (26,430, 70%) and testing (11,328, 30%) cohorts. There were 22,637 (59.95%) male and 15,121 (40.05%) female patients. The mean age was 63.16 years, and almost all of the patients (36,288, 96.11%) were white. Most of these patients were in the T1 (24,016, 63.61%), N0 (32,692, 86.58%), and M0 (34,376, 91.04%) stages. The longest follow-up period was 191 months, and the median was 85.03 months. CMM caused 5,895 deaths (15.61%). The survival curves and fundamental clinical data did not differ significantly between the two cohorts. Table 1 lists the fundamental features of the two patient groups, and Figure 3 displays the Kaplan–Meier analysis curve.

**Table 1:** Baseline characteristics of Cutaneous Malignant Melanoma.

Variable	Total *N* (%)	Train cohort *N* (%)	Test cohort *N* (%)	*p*
Patients	37,758	26,430(70%)	11,328(30%)	
Age				0.281
mean ± sd	63 ± 16.23	63 ± 16.20	63 ± 16.30	
CS_tumor_size^a^				0.528
Mean ± SD	10 ± 167.05	10 ± 165.79	10 ± 169.94	
CS_extension^b^				0.129
Mean ± SD	300 ± 232.36	300 ± 232.91	300 ± 231.07	
Surg_Prim_Site^c^				0.132
Mean ± SD	31 ± 13.42	31 ± 13.44	31 ± 13.38	
Sex				0.133
Male	22,637 (59.95%)	15,780 (59.70%)	6,857(60.53%)	
Female	15,121(40.05%)	10,650(40.30%)	4,471(39.47%)	
Race				0.812
White	36,288(96.11%)	25,402(96.11%)	10,886(96.1%)	
Black	223(0.59%)	160(0.61%)	63(0.56%)	
Other	1,247(3.3%)	868(3.28%)	379(3.35%)	
Marital status				0.922
Married	21,073(55.81%)	14,733(55.74%)	6,340(55.97%)	
Single	4,958(13.13%)	3,476(13.15%)	1,482(13.08%)	
Other	11,727(31.06%)	8,221(31.10%)	3,506(30.95%)	
Primary_Site				0.923
HF^d^	7,139(18.91%)	4,984(18.86%)	2,155(19.02%)	
Truck	15,377(40.73%)	10,765(40.73%)	4,612(40.71%)	
Limbs	15,242(40.37%)	10,681(40.41%)	4,561(40.26%)	
T				0.208
T0	3,318(8.79%)	2,344(8.87%)	974(8.60%)	
T1	24,016(63.61%)	16,722(63.27%)	7,294(64.39%)	
T2	5,004(13.25%)	3,499(13.24%)	1,505(13.29%)	
T3	2,844(7.53%)	2027(7.67%)	817(7.21%)	
T4	2,212(5.86%)	1,584(5.99%)	628(5.54%)	
TX	364(0.96%)	254(0.96%)	110(0.97%)	
N				0.930
N0	32,692(86.58%)	22,893(86.62%)	9,799(86.50%)	
N1	2,297(6.08%)	1,599(6.05%)	698(6.16%)	
N2	857(2.27%)	594(2.25%)	263(2.32%)	
N3	636(1.68%)	441(1.67%)	195(1.72%)	
NX	1,276(3.38%)	903(3.42%)	373(3.29%)	
M				0.961
M0	34,376(91.04%)	24,067(91.06%)	10,309(91.00%)	
M1	3,378(8.95%)	2,360(8.93%)	1,018(8.99%)	
MX	4(0.01%)	3(0.01%)	1(0.01%)	
Summary_Stage				0.964
Localized	30,646(81.16%)	21,443(81.13%)	9,203(81.24%)	
Regional	3,591(9.51%)	2,520(9.53%)	1,071(9.45%)	
Distant	3,521(9.33%)	2,467(9.33%)	1,054(9.30%)	
Surgery				0.209
Yes	33,757(89.40%)	23,595(89.27%)	10,162(89.71%)	
No	4,001(10.60%)	2,835(10.73%)	1,166(10.29%)	
Radiation				0.696
Yes	1865(4.94%)	1,313(4.97%)	552(4.87%)	
No	35,893(95.06%)	25,117(95.03%)	10,776(95.13%)	
Chemotherapy				0.277
Yes	1,533(4.06%)	1,054(3.99%)	479(4.23%)	
No	36,225(95.94%)	25,376(96.01%)	10,849(95.77%)	
Reg_LN_Sur^e^				0.624
Yes	11,975(31.72%)	8,362(31.64%)	3,613(31.89%)	
No	25,783(68.28%)	18,068(68.36%)	7,715(68.11%)	
Rad_Seq^f^				
before	44(0.12%)	30(0.11%)	14(0.12%)	
after	1,207(3.20%)	852(3.22%)	355(3.13%)	
Intraoperative	3(0.01%)	3(0.01%)	0(0%)	
both	28(0.07%)	19(0.07%)	9(0.08%)	
No	36,476(96.6%)	25,526(96.58%)	10,950(96.66%)	
Income				0.941
low	5,680(15.04%)	3,983(15.07%)	1,697(14.98%)	
mediate	17,430(46.16%)	12,186(46.11%)	5,244(46.29%)	
high	14,648(38.79%)	10,261(38.82%)	4,387(38.73%)	
Status				0.618
Death	5,895(15.61%)	4,101(15.52%)	1794(15.84%)	
Alive	31,863(84.39%)	22,329(84.48%)	9,534(84.16%)	

a CS tumor size: Information on tumor size. Available for after 2004 year. Earlier cases may be converted and new codes added which were not available for use prior to the current version of CS.

b CS extension: Information on extension of the tumor. Available for after 2004 year. Earlier cases may be converted and new codes added which were not available for use prior to the current version of CS.

c Surg_Prim_Site: Surgery of Primary Site describes a surgical procedure that removes and/or destroys tissue of the primary site performed as part of the initial work-up or first course of therapy.

d HF: head and face.

e Reg_LN_Sur: Scope of Regional Lymph Node Surgery describes the procedure of removal, biopsy, or aspiration of regional lymph nodes performed during the initial work-up or first course of therapy at all facilities.

f Rad_Seq: This field records the order in which surgery and radiation therapies were administered for those patients who had both surgery and radiation.

### Variable screening and DeepSurv model training

**Figure 4:**
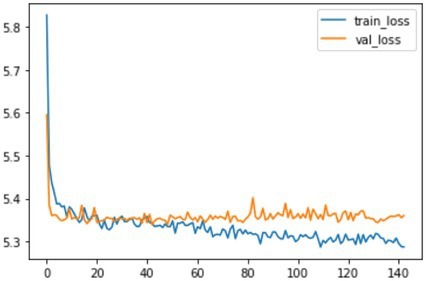
The loss change process diagram of training and validating. train_loss: train loss; Val_loss: validation losss. Train loss is the loss on the training data, which measures the fitting ability of the model on the training set. Val loss is the loss on the validation set, which measures the fitting ability on unseen data.

Using the CoxPH model to analyze the multivariate factors of the training cohort revealed that the risk factors affecting patient death were age, sex, marital status, summary stage, surgery, radiotherapy, chemotherapy, postoperative lymph node dissection, tumor size, and tumor extension (Table 2). The C-index of the CoxPH model was 0.875. We constructed the DeepSurv model using the training cohort, and the C-index of this model was calculated as 0.910. Figure 4 displays the training loss-function diagram. A comparison revealed that the DeepSurv model performed more effectively.

**Table 2:** Survival predictors in Cox PH model.

Variables	β	HR	95%CI	*p*
Age	0.03	1.03	1.03–1.04	<0.001***
Female	−0.33	0.72	0.65–0.80	<0.001***
Single	0.35	1.43	1.24–1.64	<0.001***
Marital other	0.21	1.23	1.10–1.38	<0.001***
N1	0.13	1.14	0.96–1.34	0.124
N2	0.38	1.47	1.17–1.86	0.001**
N3	0.67	1.97	1.57–2.47	<0.001***
NX	0.42	1.53	1.31–1.80	<0.001***
M1	1.77	5.85	3.41–10.05	<0.001***
Summary_Stage R	1.52	4.59	3.77–5.57	<0.001***
Summary_Stage D	1.05	2.85	1.64–4.96	<0.001***
Surgery NO	−0.33	0.72	0.58–0.87	<0.001***
Radiation NO	−0.37	0.69	0.61–0.78	<0.001***
Chemotherapy NO	−0.31	0.73	0.64–0.84	<0.001***
Reg_LN_Sur NO	−0.24	0.79	0.70–0.89	<0.001***
CS_tumor_size	0.01	1.01	1.00–1.06	0.035*
CS_extension	0.01	1.01	1.00–1.07	<0.001***

Summary_Stage R, Summary Stage in Regional; Summary_Stage D, Summary Stage in Distant; Surgery NO, NO Surgery; Chemotherapy NO, NO Chemotherapy; Reg_LN_Sur NO, NO Regional Lymph Node dissection after Surgery.

### Comparison of DeepSurv model and CoxPH model in the testinh cohort

**Figure 5:**
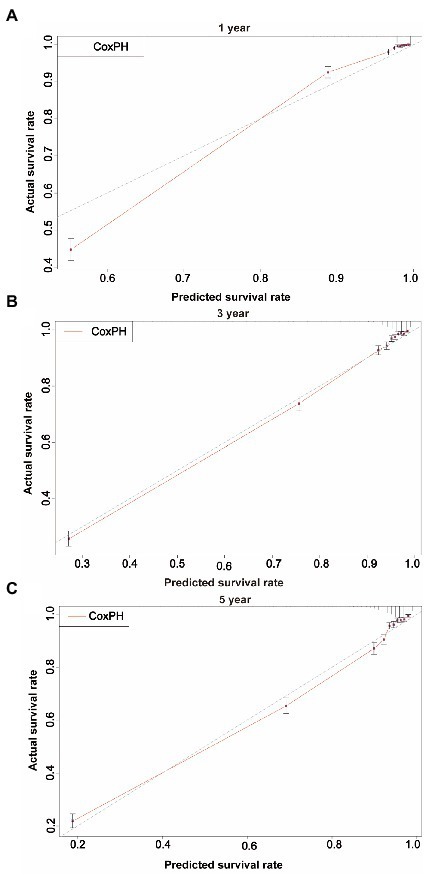
Calibration plots of survival rate of CMM in Cox PH model.

**Figure 6:**
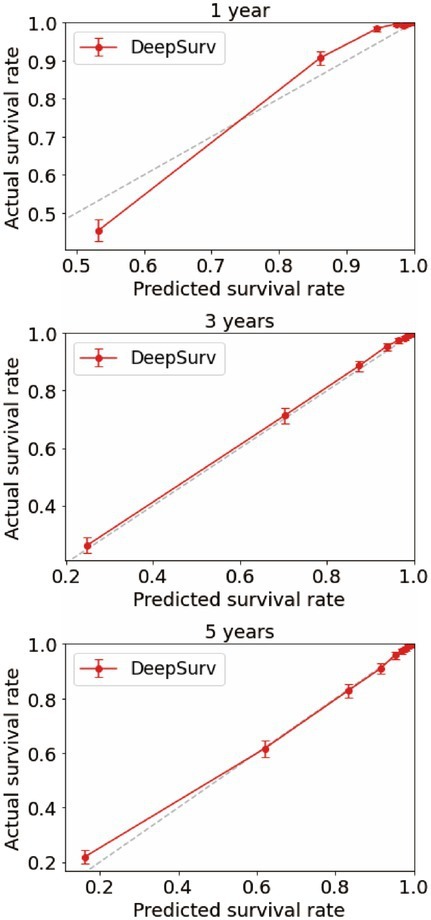
Calibration plots of survival rate of CMM in DeepSurv model.

**Figure 7:**
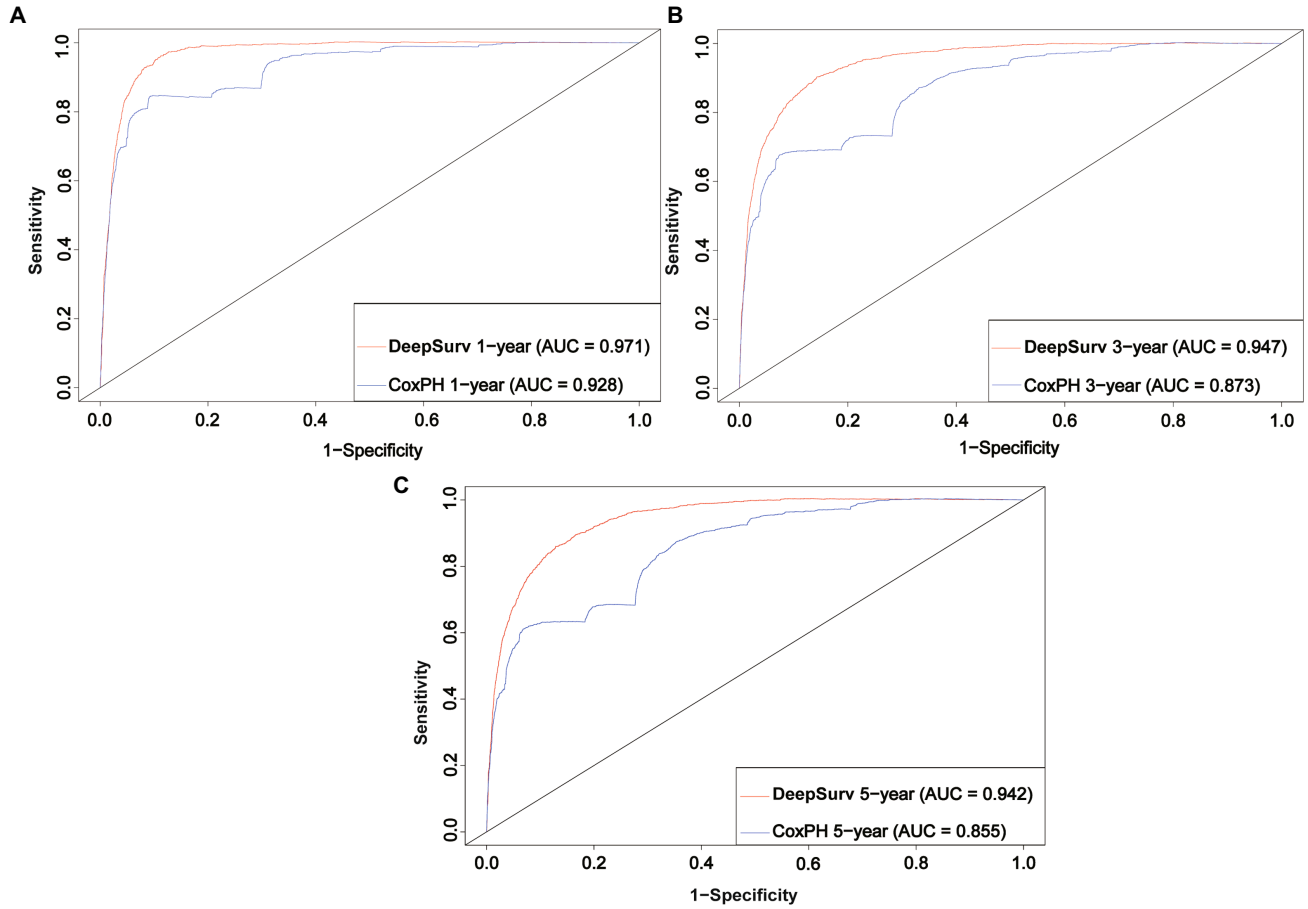
ROC curves. Comparison of ROC between the CoxPH model and the DeepSurv model in 1 year **(A)**, 3 year **(B)**, and 5 year **(C)**.

We first constructed calibration curves for patients with CMM at 1, 3, and 5 years to verify the accuracy of the CoxPH (Figure 5) and DeepSurv (Figure 6) models in predicting survival probability. The discrimination between the two models was then evaluated by plotting the ROC curves of patients with CMM at 1, 3, and 5 years and calculating the time-dependent area under the ROC curve (AUC) value (Figure 7). The results indicated that the 1-, 3-, and 5-year AUCs of the DeepSurv model (0.971, 0.947, and 0.942, respectively) were all higher than those of the Cox model (0.928, 0.837, and 0.855, respectively). These results indicate that the DeepSurv model had better discrimination and calibration abilities than the CoxPH model in predicting the survival prognosis of patients with CMM.

## Discussion

There are more than 232,100 (1.7%) new cases of CMM identified each year worldwide, and there are roughly 55,500 (0.7%) new cases of CMM-related mortality. However, there are significant regional and population-based variations in the morbidity and death rates of CMM, which may be due to variations in primary care, early identification, and treatment approaches (24). However, the survival rate for melanoma plummets after metastasis occurs (25). Although overall survival has increased over the past 10 years thanks to the development of medications such as immunosuppressants, the mortality rate remains high in patients with severe CMM (26–28).

Mortality among CMM patients varies significantly by country of residence, race, and economic status. Therefore, in addition to the treatment options, other variables should also be considered, such as the environment around the patient, the mood of the patient, etc. Only in this way can the survival rate of the patient be improved. Many survival prediction models have been developed to increase the precision of patient survival-time predictions (29–33). CoxPH is currently the most frequently employed model, but its accuracy is restricted (34) due to the linearity of its factors. The DeepSurv method is being applied in various clinical medicine subspecialties. Numerous studies have indicated that it outperforms conventional linear prediction models in survival predictions (35–37). Previous studies have demonstrated that the DeepSurv model can predict survival rates and times more accurately than can the conventional CoxPH model. For instance, it has been demonstrated that the DeepSurv model is more accurate than the CoxPH model in predicting the survival prognoses of patients with lung cancer, colon adenocarcinoma, and Coronary Care Units (38–40).

In this study, 70% of all patients with CMM were assigned to the training cohort for the multivariate analysis of the CoxPH model and to establish the DeepSurv model. The remaining 30% were allocated to the test cohort, which was used to verify the prediction abilities of the two models. Age, sex, marital status, summary stage, surgery, radiotherapy, chemotherapy, postoperative lymph node dissection, tumor size, and tumor extension were all identified as risk factors for CMM by the CoxPH model (Table 2). The C-index of the CoxPH model was 0.875. The newly established DeepSurv model has a seven-layer neural network, and its C-index was 0.910. We also found that the calibration curve of the DeepSurv model was more evenly distributed and closer to the leading-diagonal line than that of the CoxPH model, and its AUC curve was also smoother when we used the test cohort to verify the factors influencing 1-, 3-, and 5-year mortality and survival-time predictions for patients with CMM. Moreover, the AUC curve was higher than that of the CoxPH model, which indicated that the prediction and discrimination abilities of the DeepSurv model are superior to those of the CoxPH model. Because it uses multilevel neural networks to address issues such as large samples, multiple variables, and nonlinearity, the DeepSurv model has important advantages in predicting survival prognosis over other models.

This study had several limitations. First, important factors that affect prognosis, including surgery techniques, radiotherapy procedures and doses, chemotherapy types, medications, and other details, were missing from the data of patients with CMM gathered from the SEER database. Second, without external validation, the data derived from our research solely consisted of information from a few states of the US. In our future work we will further improve the DeepSurv model using richer data from more regions. Third, the hidden layer of the DeepSurv model is opaque when carrying out computational tasks, hence acting as a “black box” that prevented us from fully describing the prediction process of the model and comprehending how it makes judgments. We plan to address the above-mentioned issues in our next study.

## Conclusion

This study was the first to develop a DeepSurv prediction model specifically for accurately predicting the survival time of patients with CMM. The DeepSurv model has considerable advantages over the CoxPH model in predicting the factors affecting patient survival. The DeepSurv model can be employed as a novel analytical tool to predict outcomes and suggest treatments for patients with CMM.

## Data availability statement

The datasets presented in this study can be found in online repositories. The names of the repository/repositories and accession number(s) can be found in the article/Supplementary material.

## Author contributions

HY, LD, and JL take responsibility for the integrity of the data and the accuracy of the data analysis. WY and HY: concept and design. HY, SW, LW, and SX: acquisition, analysis, or interpretation of data. HY: drafting of the manuscript. XX, QZ, and W-kM: statistical analysis. SX and JL: administrative, technical, or material support. YH: Revise. YH, LD, and JL: supervision. All authors had full access to all of the data in the study. All authors had critical revision of the manuscript for important intellectual content. All authors contributed to writing of the manuscript and approved the final version.

## Funding

This research was funded by Key Scientific Problems and Medical Technical Problems Research Project of China Medical Education Association (grant number 2022KTZ009) and Guangdong Provincial Key Laboratory of Traditional Chinese Medicine Informatization (grant number 2021B1212040007).

## Conflict of interest

The authors declare that the research was conducted in the absence of any commercial or financial relationships that could be construed as a potential conflict of interest.

## Publisher’s note

All claims expressed in this article are solely those of the authors and do not necessarily represent those of their affiliated organizations, or those of the publisher, the editors and the reviewers. Any product that may be evaluated in this article, or claim that may be made by its manufacturer, is not guaranteed or endorsed by the publisher.
